# Antihypertensive prescription among black patients without compelling indications: prescription, effectiveness, quality and cost of medication

**DOI:** 10.1186/s12913-019-4202-2

**Published:** 2019-06-13

**Authors:** Onyinye Onyeka Akunne, Aduragbenro Deborah A. Adedapo

**Affiliations:** 1 Department of Pharmacology and Therapeutics, University of Ibadan, Ibadan, Nigeria; 2 Department of Clinical Pharmacology, University College Hospital, Ibadan, Nigeria

**Keywords:** Prescription pattern, High blood pressure, Daily defined doses, Cost, Nigeria

## Abstract

**Background:**

Hypertension remains one of the leading causes of death in Nigeria. Appropriate and cost-effective treatment of the disease is necessary to reduce mortality. This study evaluates (i) the prescription patterns and quality (ii) blood pressure control and (iii) cost of medication among patients with hypertension uncomplicated by co-morbid diseases or compelling indications.

**Method:**

Patients with uncomplicated hypertension attending three clinics in the University College Hospital, Ibadan in Nigeria were recruited into this study. Information on demographics, antihypertensive medication prescribed, blood pressure measurements, and cost of medications were collected for each patient. Antihypertensive medications were classified according to the Anatomical Therapeutic Chemical (ATC) classification system and the Defined Daily Dose (DDD) system. The frequency of usage of each drug class and their prescribed doses per patient/day were calculated and compared with the DDD to assess the quality of prescription. Cost of antihypertensive medication was calculated for each patient and reported as cost per patient/day and cost per patient/month. Effect of variables on BP control was ascertained. Statistical analyses were done using SPSS, chi-square and correlation test was used to test for associations.

**Result:**

A total number of 1050 hypertensive patients were included in this study. The mean age was 60 years, females made up 62% of the study population. A high level of polypharmacy (87%) and sub-optimal blood pressure control was observed. An increase in blood pressure was observed with increase in the number of medication prescribed (χ^2^ = 33.618, *p* < 0.001; *r* = .18, *p* < 0.001). The most prescribed antihypertensive medication either as a single therapy or a fixed-dose combination was diuretic. About 54% of the prescribed daily doses of antihypertensive medication exceeded the DDD. The total monthly expenditure on antihypertensive drugs was approximately N3.2 million ($15,300).

**Conclusion:**

Study findings show a high level of polypharmacy and non-generic prescribing. Increased prescribing of drugs that are cost-effective, as well as prescription of fixed dose combinations (FDCs), is recommended in hypertensive patients. This is necessary to control blood pressure while increasing treatment adherence.

## Background

Hypertension is a global cause of morbidity and mortality [1]. A large proportion of individuals affected with hypertension are unaware of their status [2]. Previously thought to be a disease of middle and old age; current studies suggest that incidence of this disease in the younger adults is on a rise [3]. Hypertension is frequently complicated by other diseases [4]. In patients with uncomplicated hypertension, stringent blood pressure lowering measures together with adequate monitoring is required. This will prevent complications that may lead to end-organ damage.

The quality of antihypertensive medication prescribed is vital to successful management of hypertension. Quality of prescription takes into consideration the rationality of medication use among patients [5]. Medicine use is rational, proper and correct when patients receive the appropriate medicines, in doses that meet their own individual requirements. This should be for an adequate period of time, and at the lowest cost both to the patients and the community [5]. Conversely, irrational use of medicines is when one or more of these stipulated conditions are not met [6]. Guidelines on appropriate medication and dosing for treatment of uncomplicated hypertension based on multiple clinical trials are available [7–9]. These guidelines are provided to promote rational use and prescribing of medications. Although all classes of antihypertensive medication have been shown to be comparable in lowering blood pressure, thiazide-type diuretics and calcium channel blockers (CCBs) are recommended as first-line treatment for uncomplicated hypertension in blacks [7]. Thiazide-type diuretics and CCBs are more effective in improving cerebrovascular, heart failure, and combined cardiovascular outcomes compared to an angiotensin converting enzyme inhibitors (ACEI) in blacks [7]. CCBs are less effective in preventing heart failure among black hypertensives when compared with diuretics but they are more effective in reducing BP in blacks than ACEI [7]. Prescription patterns of antihypertensive medication vary by region in Nigeria, however, prescriptions of CCBs have increased in recent times [10]. A 10-year trend analysis performed by Eishet and Yusuff showed a sharp decline in the prescription of diuretics from 39.4 to 16.1% [11]. Thiazide-diuretics are cost-effective and prescription of this medication will reduce patients’ out-of-pocket expenses [12]. Quality of prescription, including conformation to stipulated guidelines and correct dosing, is necessary to improve clinical outcomes in hypertensive patients. The acquisition cost of medication is vital to the management of hypertension [13]. This is especially true in Nigeria where most patients pay out of pocket for their medication [14]. The cheapest medication proven to have a significant positive effect on health outcomes should be prescribed to reduce expenditure on medication.

Previous studies carried out on prescription quality included a limited number of patients [10, 11] and the doses of antihypertensive medication prescribed by physicians were not studied. High rates of under dosing of medication have been noted in Nigeria [15]. Very few studies previously conducted in this region considered medication cost [12, 16]. Reduced cost of treatment will improve patient’s adherence and clinical outcomes [13]. This study examines (i) the prescription patterns and quality (ii) blood pressure control and the effect of mono- or combination therapy (iii) cost of medication among patients with hypertension uncomplicated by comorbid diseases or compelling indications.

## Method

Hypertensive patients without compelling indications attending three clinics of the University College Hospital, Ibadan, Nigeria were included in the study. This cross-sectional study was conducted over a period of 6 months from November 2011 to April 2012. Patients above 30 years receiving treatment (including life style modification) for hypertension were included in the study. Patients with comorbid conditions such as diabetes mellitus, renal diseases and/or complications such as congestive heart failure, Ischemia were excluded from the study. Patient recruitment and enrolment was by convenient sampling. Medical records of consecutive patients were evaluated for completeness. Patient records containing more than 80% of information required for analysis were included in the study. The most recent information about patients was used for analysis. Patients’ age, sex, BMI, antihypertension medication prescribed with dosage and frequency of administration and blood pressure readings were collected from their records.

Antihypertensive drugs prescribed were evaluated for quality and conformity with current guidelines. Drugs were classified according to the Anatomical Therapeutic Chemical classification (ATCC) and daily defined dose (DDD) system [17]. Antihypertensive medications were classified into 13 groups. The groups were- Centrally Acting Anti-adrenergic Agents (C02AB), Low-Ceiling Thiazides Diuretics (C03AA), Low-Ceiling Diuretics excl. Thiazides (C03B), High-Ceiling Diuretics (C03CA), Potassium-Sparing Agents (C03DA), Low-Ceiling Diuretics and Potassium-Sparing Agents (C03E), Beta Blocking Agents (C07A), Beta Blocking Agents and Thiazides (C07BB), Calcium Channel Blockers (C08CA), Angiotensin converting enzyme (ACE) Inhibitors (C09AA), ACE inhibitors and diuretics (C09BA), Angiotensin II antagonists (C09CA), and Angiotensin II Antagonists Combinations with thiazide diuretic and/or calcium channel blockers (C09D). Prescribed doses were classified in relation to the DDD as (i) prescriptions with doses greater than the recommended DDD (ii) prescriptions containing less than the recommended DDD (iii) Prescriptions containing doses equal to recommended DDD and (iv) prescriptions for which doses were not available. Blood pressure threshold of 140/90 was used to classify blood pressure control in patients. Patients with blood pressure reading < 140/90 were grouped as patients with blood pressure controlled while those with blood pressure reading ≥140/90 were grouped as patients with uncontrolled blood pressure. Recommendations stipulate that at least two blood pressure readings of patient be taken at each visit [9]. In instances where the two blood pressure readings were available for a patient at one clinical visit, the mean blood pressure reading was used. The effect of centrally acting adrenergic drugs, beta blockers, calcium channel blockers, renin-angiotensin medication (Angiotensin-converting enzyme inhibitor and angiotensin receptor blocker) and diuretics prescribed as either mono-or combination therapy was assessed in the groups with controlled and uncontrolled blood pressure. The total expenditure on antihypertensive medication, average cost of medication per patient per day and average cost of medication per month was calculated in Naira and converted to the Dollar equivalent based on the conversion rate at the time the study was conducted (N200 ≈ 1USD).

SPSS statistical package version 24 was used for analysis. Socio-demographic and clinical variables of patients was presented as frequencies and percentage. Chi-square test was used to test for significance between the groups of variables (sex, age, BMI, blood pressure control, number of antihypertensive medication prescribed). The effect of number of medication prescribed on blood pressure control was also tested by chi-square. Correlation between blood pressure control and age, sex, BMI and the number of antihypertensive medication prescribed was evaluated.

## Results

A total of 1050 hypertensive patients above 30 years of age receiving treatment for hypertension without complications or comorbid conditions were enrolled into the study. The female to male ratio was 1:0.63 (*F* = 646 (61.5%), M = 404 (38.5%)). The mean age of patients was 59.9 years ±12.1 (range = 30–85), with most patients above 59 years. Blood pressure control was poor with only 35% of the patients achieving optimum blood pressure values (Table 1).

**Table 1:** Socio-demographic/Clinical variables of hypertensive Patients attending Clinics at the University College Hospital Ibadan, Nigeria

Variable		Frequency (*n* = 1050)	Percentage (%)
Sex^a^			
	Female	646	61.5
	Male	404	38.5
Age^a^			
	30–39	68	6.5
	40–49	135	12.9
	50–59	256	24.4
	60–69	323	30.8
	70–79	231	22.0
	≥80	37	3.5
BMI^a^			
	<18.5	49	4.7
	18.5–24.9	364	34.7
	25.0–29.9	334	31.8
	≥30	303	28.9
No. of antihypertensive drugs prescribed			
	0	19	1.8
	1	122	11.6
	2	445	42.4
	3	369	35.1
	4	85	8.1
	5	10	1.0
No. of fixed dose combination containing two or more classes of antihypertensive drugs prescribed			
	2	61	5.8
	3	4	0.4
Blood Pressure Control^a^			
	SBP/DBP controlled	369	35.1
	SBP/DBP uncontrolled	381	36.3
	Only SBP controlled	153	14.6
	Only DBP controlled	140	13.3
	Missing data	7	0.7

^a^χ^2^, *P* < 0.0005

### Prescription pattern and quality

Patients had as many as 5 antihypertensive drugs prescribed; however, 19 patients were on non-pharmacological therapy. The total number of antihypertensive drugs prescribed was 2504. Fixed dose combinations containing at least two different classes of antihypertensive drugs were prescribed to 65 patients. Most patients received prescription containing more than one antihypertensive (Table 1), the average number of antihypertensive medication prescribed was of 2 ± 1.

Diuretics were the most prescribed drug class singly or in combination with other drug class (33.9%) (Table 2), beta-blockers (5.0%) were the least prescribed drug class. The prescribed daily dose in 54.0% of drugs prescribed exceeded the defined daily dose, while 22.3% of antihypertensive medication prescribed had doses below the defined daily dose.

**Table 2:** Comparison between Prescribed Daily Dose (PDD) and Daily Defined Dose (DDD)

Drug Class/ATCC Code	PDD > DDD	PDD < DDD	PDD = DDD	Dose Un-available	Total (%)
Centrally Acting anti-adrenergic drugs, C02AB	60	133	63	2	258 (10.3)
Low-Ceiling Diuretics (Thiazides), C03AA	20	34	66	1	121 (4.8)
Low-Ceiling Diuretics excl. Thiazides, C03B	5	3	4	0	12 (0.5)
High-Ceiling Diuretics, C03CA	11	5	15	0	31 (1.2)
Potassium-Sparing Agents, C03DA	2	36	1	0	39 (1.6)
Low-Ceiling Diuretics and Potassium-Sparing Agents, C03E	515	0	56	1	572 (22.8)
Beta Blocking Agents, C07A	28	92	3	1	124 (5.0)
Beta Blocking Agents and Thiazides, C07BB	2	1	0	0	3 (0.1)
Calcium Channel Blockers, C08CA	497	70	153	6	726 (29.0)
Ace Inhibitors, C09AA	155	167	141	3	466 (18.6)
ACE inhibitors and diuretics, C09BA	3	1	18	0	22 (0.9)
Angiotensin II antagonists, C09CA	32	14	41	0	87 (3.5)
Angiotensin II Antagonists (Combination with thaizide diuretic and/or calcium channel blockers), C09D	22	2	18	1	43 (1.7)
Total (%)	1352 (54.0)	558 (22.3)	579 (23.1)	15 (0.6)	2504 (100)

### Blood pressure control

The proportion of patients with uncontrolled blood pressure increased with increasing number of antihypertensive medication prescribed (Table 3). The number of drugs prescribed positively correlated with systolic blood pressure (*r* = .25, *p* < 0.001, *n* = 1043) and diastolic blood pressure (*r* = .18, *p* < 0.001, *n* = 1043). Age also correlated with blood pressure control (*r* = .17, *p* < 0.001, *n* = 1043). There was no correlation between blood pressure control and sex of patients.

**Table 3:** Effect of mono-combination therapy on Blood pressure control

Number of antihypertensive medication prescribed	Number of patients with BP controlled (%)	Number of patients with BP uncontrolled (%)	Patients with BP Measurements missing (%)	Total (%)	*P*-Value
0	11 (57.9)	8 (42.1)	–	19 (100)	0.000
1	62 (50.8)	59 (48.4)	1 (0.8)	122 (100)	
2	164 (36.9)	278 (62.5)	3 (0.67)	445 (100)	
3	115 (31.2)	251 (68.0)	3 (0.8)	369 (100)	
4	16 (18.8)	69 (81.2)	–	85 (100)	
5	1 (10.0)	9 (90.0)	–	10 (100)	
Total	369 (35.1)	674 (64.2)	7 (0.7)	1050 (100)	

*χ^2^ = 33.618 df = 5, *p* < 0.001

### Acquisition cost of antihypertensive medication

Cost analysis of prescriptions showed that the total expenditure by study participants on antihypertensive medication per day was N 103,845.75_o103,845.8 ($519.4) (Table 4). The average cost per patient per day was ₦100.7 ($0.50). Patients’ daily expenditure on medication ranged from ₦5 ($0.03) to ₦817 ($4.09). Diuretics were the cheapest drug class prescribed as a monotherapy while centrally acting adrenergic drugs were the most expensive medication prescribed. The average cost of medication per patient per month was ₦3021.7 ($14.8). Patients spent between ₦150 ($0.75) and ₦24,510($122.55) monthly on antihypertensive medication. The total of monthly expenditure on antihypertensive medication was ₦3,115,372.5 ($15,292.95). The most prescribed drug combination was CCB, renin-angiotensin system drug (ACEI or ARB) and diuretics. The least prescribed drug combination was a centrally acting adrenergic drug, beta-blocker, CCB and renin-angiotensin system drug.

**Table 4:** Cost of Antihypertensive Medication Prescribed to Patients

Drug Prescribed/ATCC Code		No. of patients	Total cost/ day ^a^ ₦($)	Average cost/patient/day ^a^₦($)	Total cost/month ^a^₦($)	Average cost/patient/month ^a^₦($)
CAAD only		2	270 (1.4)	135 (0.68)	8100 (40.5)	4050 (20.25)
CAAD with	BB	3	2.2 (449)	149 (0.75)	13,470 (67.4)	4470 (22.35)
	BB, CCB	2	463.5 (2.3)	231 (1.16)	13,905 (69.5)	6930 (34.65)
	BB, CCB, R-A	1	354 (1.8)	354 (1.77)	10,620 (53.1)	10,620 (53.10)
	BB, CCB, R-A,D	6	4907 (24.5)	817 (4.09)	147,210 (736.1)	24,510 (122.55)
	BB, CCB, D	5	1292 (6.5)	258 (1.29)	38,760 (193.8)	7740 (38.70)
	BB, R-A, D	2	556 (2.8)	278 (1.39)	16,680 (83.4)	8340 (41.70)
	BB, D	4	614 (3.1)	153 (0.77)	18,420 (92.1)	4590 (22.95)
	CCB	19	2282 (11.4)	120 (0.60)	68,460 (342.3)	3600 (18.00)
	CCB, R-A	31	6760 (33.8)	218 (1.09)	202,800 (1014)	6540 (32.70)
	CCB, R-A, D	54	14,051 (70.3)	260 (1.30)	421,530 (2107)	7800 (39.00)
	CCB, D	69	14,896 (74.5)	215 (1.08)	446,880 (2234.4)	6450 (32.25)
	R-A	7	1119 (5.6)	159 (0.80)	33,570 (167.9)	477 (2.39)
	R-A,D	27	5336 (26.7)	197 (0.99)	160,080 (800.4)	5910 (29.55)
	D	31	4044 (20.2)	130 (0.65)	121,320 (606.6)	3900 (19.50)
BB only		3	997.5 (5.0)	332 (1.66)	29,925 (141.8)	9960 (49.80)
BB with	CCB	9	407 (2.0)	45 (0.23)	12,210 (61.1)	1350 (6.75)
	CCB, R-A	9	934 (4.7)	103 (0.52)	28,020 (140.1)	3090 (15.45)
	CCB, R-A,D	19	2101 (10.5)	110 (0.55)	63,030 (315.2)	3300 (16.50)
	CCB, D	14	627.3 (3.1)	44 (0.22)	18,817.5 (94.1)	1320 (6.60)
	R-A	5	244 (1.2)	48 (0.24)	7320 (36.6)	1440 (7.20)
	R-A,D	21	7.6 (516.5)	72 (0.36)	45,495 (227.5)	2160 (10.80)
	D	14	235 (1.2)	16 (0.08)	7050 (35.3)	480 (2.40)
CCB only		36	840.5 (9.21)	51 (0.26)	55,215 (0.3)	1530 (7.65)
CCB with	R-A	86	4738 (23.7)	55 (0.28)	142,140 (710.7)	1650 (8.25)
	R-A, D	207	18,602 (93.0)	89 (0.45)	558,060 (2790.3)	2670 (13.35)
	D	168	5845.5 (29.2)	34 (0.17)	175,365 (876.8)	1020 (5.10)
R-A only		31	1003 (5.0)	32 (0.16)	30,090 (150.5)	960 (4.80)
R-A with	D	104	7111 (35.6)	68 (0.34)	213,330 (1066.7)	2040 (10.20)
D only		42	250 (1.3)	5 (0.03)	7500 (37.5)	150 (0.75)
TOTAL		1031	103,845.75 (519.4)		3,115,372.5 (15,292.95)	

^a^1 USD ≈ 200 NGN. *CAAD* Centrally Acting Anti-adrenergic Drug, *BB* Beta Blockers, *CCB* Calcium Channel Blockers, *R-A* Renin-Angiotensin system drug, *D* Diuretics, *NA* not available

## Discussion

This study reveals inadequate blood pressure control among study participants. Moreover, we found use of multiple medicines (poly-pharmacy) was commonly practiced in the management of hypertension. In Nigeria majority of people with hypertension are between 40 and 60 years [16, 18, 19], this is also reflected in this study as most of the patients were between 40 and 69 years with the average age being 60 years. A survey in the United States [20] showed that the incidence of hypertension is greater in those aged above 60 years. The study also showed proportionality in the male to female ratio of individuals with hypertension in this age group compared to patients younger than 60 years where men were more likely to be hypertensive. In our study, the women to men ratio was high. Stratification according to race shows that hypertension is more likely to occur in black women than non-black women [21].

Lifestyle modifications are recommended as the first line of therapy in hypertension especially in black patients without compelling indications or co-morbid diseases [22]. Where these methods fail to achieve BP goals, antihypertensive medication can be initiated in patients. In our study, 1.8% of participants were on lifestyle modification to control blood pressure. Prescription of more than one class of antihypertensive was high. This agrees with study findings by Gu et al., 2017 [23], in their study they found that blacks had more aggressive forms of hypertension and were more likely to receive combination therapy to achieve optimum blood pressure goals. A foremost recent randomized controlled trial of antihypertensive drug combinations among black sub-Saharan Africans suggested that CCB in combination with diuretics or ACEIs was more effective than non-CCBs combination [24]. Poly-pharmacy can lead to poor patients’ adherence to treatment [25]. Adherence to treatment is affected by high pill burden and treatment cost [26]. This can be remedied by prescription of fixed-dose combination (FDCs) antihypertensive drugs. Studies carried out by Verma et al and Maza et al suggests that prescription of FDCs improves patients’ adherence to treatment leading to better clinical outcomes [27, 28]. FDCs have also been shown to be more efficacious in the treatment of hypertension among blacks [29]. Only a few patients received fixed-dose combinations. Prescribing FDCs should be encouraged to improve clinical outcomes. 

In blacks, diuretics and CCBs have been shown to reduce BP more effectively than ACEIs, ARBs and beta-blockers [7]. They are also more effective in reducing the incidence of cardiovascular diseases [7]. Race and ethnicity, however, are not the basis for excluding any class of antihypertensive agent in combination [8]. The most prescribed drug class as monotherapy was diuretics to 42 patients followed by CCBs to 36 patients. This agrees with a previous study on patterns of monotherapy prescription [30]. More than a third of patients on monotherapy were prescribed renin-angiotensin blockers. The rationality of prescribing these medications should be taken into consideration especially as patients had no compelling indications (such as diabetes mellitus with nephropathy or heart failure). ACEIs have been shown to increase the risk of angioedema in blacks [21]. About 19% of the medication prescribed to patients was ACEI. Prescribing spironolactone in combination therapy especially in greater ≥3 drug combination reduces BP to a greater extent than including centrally acting adrenergic drugs or beta-blockers [31]. Spironolactone was minimally prescribed to patients with resistant hypertension on more than three drugs.

Daily defined dose was exceeded in more than 50% of the prescriptions. This varies with a previous report in this institution [15]. The DDD is a reference guide suggesting the optimum dose of drugs to be prescribed to patients per day [17]. Low doses of antihypertensive medication especially as combinations containing two or more drugs are advocated to reduce side effects attributable to medicine use [32]. While prescription of low doses is encouraged, the effect of under prescription should be carefully considered. Under-prescription can reduce the effectiveness of medication.

Blood pressure control was poor in this population with only about 35% of the patients achieving optimum blood pressure thresholds. Poor blood pressure control continues to be an issue in Nigeria. Previous studies carried out showed high rates of uncontrolled BP [18, 19]. Factors such as race, obesity, adherence to therapy, cost of medication and old age have been implicated in the failure to meet treatment goals among hypertensive patients [33]. The number of drugs prescribed affected blood pressure control. Increased number of drugs prescribed negatively affected blood pressure control. Almost all the patients receiving 5-drug combination and 80% of patients on 4-drug combination had inadequate blood pressure control.

Cost-effectiveness is an important determining factor in the success of therapy. Very low socio-economic status is prevalent among the Nigerian population with most people subsisting on below $1 [34]. This prevailing condition makes access to basic necessities of life such as medication and healthcare challenging especially as patients are required to pay out of pocket for medications. The highest cost of medication per patient/month in this study was $122.55 (₦24,510) and it was a 5-drug combination. This high cost of medication among patients taking 5 drugs could be a factor influencing poor BP controls observed among this group. In our study, diuretics were the most cost-effective antihypertensive treatment choice. Patients on diuretics alone paid as low as $0.75 (₦150) per patient/month. Beta-blockers were the most expensive drug prescribed as monotherapy costing a patient $49.80 (₦9960) per month.

### Study strengths

In this study, the quality of antihypertensive medications prescribed was critically evaluated. There is scarce information comparing the prescribed daily dose with the DDD. Cost implication of prescribing patterns was also studied.

### Study limitations

1. This study was carried out at clinics in one tertiary level hospital and cannot be generalized to reflect the prescribing pattern and quality in Nigeria.
2. The impact of duration of hypertension on blood pressure control was not assessed.
3. For cost consideration, comparison is a challenge because the exchange rate at the time the study was conducted is quite different from the current rates. The purchasing power parity (PPP) at the time of study was 79.8. Presently, the PPP stands at 102.7. The % change in PPP is 28.7%.

## Conclusion

Blood pressure control was low in patients. Poly-pharmacy may have contributed to the low BP control. Prescription of FDC will reduce the number of medication prescription to improve adherence to treatment. Prescribed daily dose equal to the DDD should be prescribed. This will improve clinical outcome as well as reduce adverse effects of medication. Measures to improve blood pressure control and attain acceptable BP targets should be explored in further studies.

## Data Availability

All dataset generated and analysed during the current study are available from the corresponding author on reasonable request.

## Abbreviations

ACEI: Angiotensin converting enzyme inhibitors
ARB: Angiotensin receptor blocker
ATCC: Anatomical Therapeutic Chemical classification
BP: Blood pressure
CCB: Calcium channel blocker
DDD: Defined Daily Dose
FDC: Fixed dose combinations
IRB: Institution Review Board
UI/UCH: University of Ibadan/University College Hospital
